# CDC Activities for Improving Implementation of Human Papillomavirus Vaccination, Cervical Cancer Screening, and Surveillance Worldwide

**DOI:** 10.3201/eid2313.170603

**Published:** 2017-12

**Authors:** Virginia Senkomago, Denise Duran, Anagha Loharikar, Terri B. Hyde, Lauri E. Markowitz, Elizabeth R. Unger, Mona Saraiya

**Affiliations:** Centers for Disease Control and Prevention, Atlanta, Georgia, USA

**Keywords:** Centers for Disease Control and Prevention, CDC, human papillomavirus, viruses, HPV, vaccination, cervical cancer, screening, surveillance, sexually transmitted infections, incidence rates, mortality rates, global health security

## Abstract

Cervical cancer incidence and mortality rates are high, particularly in developing countries. Most cervical cancers can be prevented by human papillomavirus (HPV) vaccination, screening, and timely treatment. The US Centers for Disease Control and Prevention (CDC) provides global technical assistance for implementation and evaluation of HPV vaccination pilot projects and programs and laboratory-related HPV activities to assess HPV vaccines. CDC collaborates with global partners to develop global cervical cancer screening recommendations and manuals, implement screening, create standardized evaluation tools, and provide expertise to monitor outcomes. CDC also trains epidemiologists in cancer prevention through its Field Epidemiology Training Program and is working to improve cancer surveillance by supporting efforts of the World Health Organization in developing cancer registry hubs and assisting countries in estimating costs for developing population-based cancer registries. These activities contribute to the Global Health Security Agenda action packages to improve immunization, surveillance, and the public health workforce globally.

Cervical cancer is one of the most commonly diagnosed cancers and a leading cause of cancer death among women worldwide; in 2012, there were an estimated 528,000 new cases and 266,000 cervical cancer deaths globally (*1*). Nearly all cervical cancers are caused by persistent infection with oncogenic human papillomavirus (HPV) types, most commonly HPV-16 and HPV-18 (*2*,*3*). Progression from persistent HPV infection to invasive cervical cancer occurs over a long period (average 7–10 years), during which cervical precancers can be detected by screening and treatment initiated to prevent invasive cervical cancer (*4*). Cervical cancer incidence is remarkably lower in North America (6.6 cases/100,000 persons) and Western Europe (7.3 cases/100,000 persons), where cervical cancer screening and treatment programs have been implemented for several decades, than in sub-Saharan Africa (34.8 cases/100,000 persons) and Latin America and the Caribbean region (21.2 cases/100,000 persons), where cervical cancer screening programs are comparatively nascent or not yet developed (Figure) (*1*,*6*).

**Figure:**
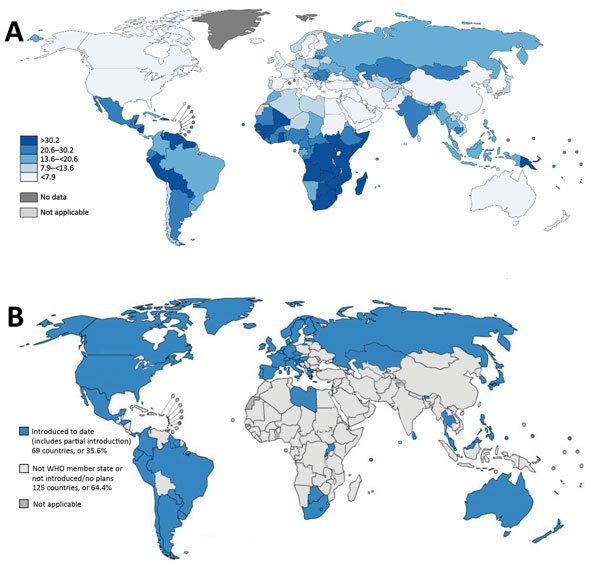
Worldwide cervical cancer incidence and human papillomavirus (HPV) vaccination status. A) Estimated cervical cancer incidence rates per 100,000 persons in 2012. Source: GLOBOCAN, 2012, WHO. B) Progress in HPV vaccine introduction in national immunization programs, 2016. Source: WHO, 2016. Many countries with high cervical cancer incidence rates (primarily countries in sub-Saharan Africa, Asia, and a few in Latin America) have not yet introduced HPV vaccination in their national immunization programs. Cervical cancer can also be prevented by screening and treatment for precancerous lesions; incidence and mortality rates in high-income countries have decreased largely because of effective screening programs. Data for cervical cancer screening coverage worldwide are limited; 2002 World Health Survey data showed that the proportion of women who had a Papanicolaou test in the previous 3 years greatly varied among countries; 11%–83% in industrialized countries, and 1%–73% in developing countries (*5*). WHO, World Health Organization.

The discovery of the strong causal relationship between persistent infections with oncogenic HPV types and cervical cancer has led to development of HPV vaccines to prevent infection by oncogenic HPV types, and HPV tests that are being used to improve cervical cancer screening. Currently, 3 HPV vaccines have been developed: a bivalent vaccine that protects against HPV-16 and HPV-18; a quadrivalent vaccine that protects against HPV-16, HPV-18, and nononcogenic HPV types 6 and 11; and a 9-valent vaccine that protects against those in the quadrivalent vaccine and 5 additional oncogenic HPV types (HPV-31, -33, -45, -52, and -58).

The World Health Organization (WHO) now recommends 2 doses of HPV vaccination for girls 9–14 years of age in all countries where cervical cancer prevention is a public health priority and introduction is feasible and sustainable. WHO also recommends vaccination of multiple age cohorts (e.g., 9–14 years) in the first year of introduction, if feasible for the country. HPV vaccination has been introduced in 71 (37%) countries worldwide; however, most developing countries with higher cervical cancer incidence rates have not yet introduced HPV vaccination (*7*). CDC is working with various partners to assist in development of global HPV vaccination policies and guidelines, and provide technical assistance in implementation and evaluation of country HPV vaccination pilot projects or programs.

Five HPV tests have been approved by the US Food and Drug Administration for clinical applications in cervical cancer screening and additional tests are available outside the United States. In 2013, WHO released new cervical cancer screening guidelines recommending the use of primary HPV testing as the preferred method in areas where effective cytologic (Papanicolaou [Pap]–based) screening programs did not already exist (*8*). CDC is working with various partners in development of cervical cancer screening guidelines and policies, and implementation and evaluation of cervical cancer screening activities.

Global cervical cancer prevention has been identified as a key actionable issue by the Council on Foreign Relations’ taskforce report on the emerging global health crisis from noncommunicable diseases (NCDs). In its report, this taskforce explains that a US focus on global NCDs, including cervical cancer prevention, would leverage and ensure the effectiveness of US global health investments that have been made in infectious disease prevention (*9*). As part of the CDC Strategic Framework for Global Immunization, during 2016–2020, the agency is committed to supporting introduction of HPV vaccine for cervical cancer prevention (*10*). Global activities of CDC in cervical cancer prevention described in this article, including providing technical assistance on HPV vaccination program introduction and laboratory assessment of HPV vaccines, training field epidemiologists, and improving cancer registration, also contribute to the Global Health Security Agenda (GHSA) action packages to improve immunization, surveillance, and workforce development. These efforts contribute to global health security by enhancing the workforce capacity of countries to rapidly detect, respond, and control public health emergencies, thereby preventing these emergencies from spreading to other countries.

## Global HPV Vaccination Activities

### Global HPV Vaccination Policies and Guidelines

Since 2005, CDC has provided technical support to WHO and its Strategic Advisory Group of Experts in the development and revision of HPV vaccine policies and guidelines. CDC participated in WHO technical work groups providing expertise for the development of the first WHO position paper and recommendations for HPV vaccination that were released in 2009 (*11*). CDC also assisted in the development of the 2010 WHO report focused on evaluating HPV vaccine coverage and providing guidance for HPV vaccine impact monitoring (*12*). In addition, CDC participated in a series of WHO meetings during 2013–2015 that were focused on revising guidelines for future HPV vaccine trials, including guidance on trial design and clinical endpoints for evaluation of new prophylactic HPV vaccines (*13*). As further data became available from vaccine trials, CDC provided ongoing consultation to WHO headquarters and the Pan American Health Organization (PAHO) when deliberations were under way for reduced-dose vaccination schedules and consideration of vaccination for boys. The Advisory Committee on Immunization Practices of CDC has provided ongoing opportunities for policy makers from other countries to attend and learn from US deliberations on vaccine policy.

### Implementation of HPV Vaccination Globally

#### Technical Assistance on HPV Vaccine Introduction

Gavi, the Vaccine Alliance, is a public–private sector partnership that funds lower-income countries for new and underused vaccines to ensure equal access of vaccines to the poorest children. CDC is a core member of the Gavi HPV Global Leadership Team, composed of international immunization partners who provide guidance in the design, implementation, monitoring, and evaluation of Gavi support for HPV vaccine introduction. In 2012, Gavi introduced its program of financial support to eligible countries for HPV vaccine introduction. Because 9–13-year-old girls were a new immunization target population for these countries, the Gavi program required demonstration of the ability to deliver vaccine to this population through smaller-scale pilot projects. Gavi provided funding for vaccine procurement, as well as operational and technical assistance with new vaccine introduction (*14*).

After 5 years of learning and experience from ≈24 country pilot projects across multiple regions and continents, the program is transitioning to catalyzing countries to scale-up of HPV vaccinations nationally, marking the first addition to national immunization programs beyond early childhood (*15*). CDC has played a key role in this transition at the global level by contributing lessons learned in pilot projects and providing feedback on the new guidelines. Gavi financially supports the introduction of HPV vaccination among Gavi-eligible countries according to the new WHO Strategic Advisory Group of Experts guidelines. These guidelines recommend targeting multiple birth cohorts of girls 9–14 years of age in the first year of vaccine introduction, if feasible for the country, followed by single-birth cohorts, to ensure maximum vaccination coverage (*16*).

CDC continues to provide technical and field support to key countries to assist with decision-making on HPV vaccine introduction, applying for financial support, implementation planning, and evaluation of vaccine introduction and programs. CDC works closely with ministries of health, WHO regional and country offices, and other immunization partners to support countries in vaccine implementation planning, including interpreting lessons learned from pilot projects, ensuring equity and coverage in delivery of vaccine, reviewing communication strategies and social mobilization planning, emphasizing the need for monitoring, and assessing financial cost of vaccine introduction. In the past 2 years, CDC has provided technical assistance to Laos, Cambodia, the Solomon Islands, Nepal, Ethiopia, Liberia, Zimbabwe, and Sierra Leone in implementing and evaluating HPV vaccine pilot projects or scale-up planning for national vaccine introduction.

#### Technical Assistance on Laboratory-Related HPV Activities

CDC has provided technical assistance to WHO and countries on assessment of HPV vaccine quality, safety, and efficacy, and on standardization of HPV testing. HPV vaccine clinical trials rely on HPV testing, both HPV serologic testing and HPV nucleic acid detection, to establish a vaccination-naive population and document biological endpoints. WHO, with support from the Bill and Melinda Gates Foundation, established the WHO HPV LabNet during 2006–2011 to assist in standardization of HPV testing. When LabNet was active, the CDC HPV Laboratory served as 1 of 2 Global Reference Laboratories. LabNet supported development of international standards for HPV DNA and HPV serologic analysis, developed proficiency testing for HPV DNA assays, and published an HPV laboratory manual, among other accomplishments. CDC has continued to support WHO initiatives related to HPV testing and HPV vaccine development through technical support in drafting the WHO technical report on the quality, safety, and efficacy of recombinant HPV virus–like particle vaccines that was released in 2015, as well in the 2 workshops held to explain the document to vaccine manufacturers and national regulatory agencies (*13*).

### Evaluation and Implementation Research on Global HPV Vaccination

CDC has supported implementation research and evaluation activities before and after HPV vaccine introduction in various countries. CDC conducted implementation research in western Kenya to assess HPV knowledge, attitudes, and beliefs to assist development of communication messages (*17*). CDC has been a technical partner in facilitation and leadership of HPV pilot project evaluations to optimize program performance; CDC has participated in or facilitated postintroduction evaluations in Laos, Ethiopia, the Solomon Islands, Cambodia, and Nepal. These program evaluations help to clarify effectiveness of program delivery, community messaging, and overall system functioning. In addition, CDC has completed coverage assessments in pilot projects in Laos, Cambodia, and Liberia to evaluate age- and dose-specific vaccination coverage among the target population, as well as assessed equitable access and vaccine acceptability. CDC also has ongoing collaborations with global immunization partners to examine and summarize HPV vaccine introduction progress worldwide, including a summary of HPV vaccination in the Americas during 2006–2010 and a global summary of HPV vaccination introduction in 39 countries in 2012 (*18**,**19*). Last, CDC economists have completed cost evaluations of the HPV pilot programs in Zimbabwe and Cambodia to estimate the financial impact of introduction of HPV vaccine and assist future scale-up planning.

## Global Cervical Cancer Screening Activities

### Global Cervical Cancer Screening Recommendations and Manuals

CDC has provided technical support and expertise in the development of global cervical cancer screening recommendations and manuals. CDC participated in the development of the second version of the WHO guidelines on cervical cancer screening and treatment, which provide resource- and HIV-stratified recommendations for implementing cervical cancer screening (*8*). WHO guidelines recommend the implementation of primary HPV screening in countries where Pap-based screening programs do not exist or are not effective. CDC partnered with PAHO in conducting policy dialogues to promote these WHO guidelines and HPV-based screening in Costa Rica, Guatemala, and El Salvador. CDC and PAHO/WHO also developed a manual to guide program managers in integrating HPV testing into cervical cancer screening programs (*20*). In addition, CDC worked collaboratively with the Union for International Cancer Control to develop a curriculum to educate nurses about HPV and cervical cancer. In many low- and middle-income countries with limited numbers of physicians, nurses play a key role in cervical cancer screening and treatment. Three workshops have been held in Central and South America to disseminate the curriculum.

### Implementation of Cervical Cancer Screening Globally

CDC is an implementing agency of the US President’s Emergency Plan for AIDS Relief (PEPFAR) and works with ministries of health to deliver sustainable HIV/AIDS prevention, care, and treatment. Given that cervical cancer incidence is higher among HIV-positive women, CDC has played a key role in convening a technical consultation on HIV and cervical cancer to support screening among HIV-positive women through the PEPFAR program. PEPFAR has provided support for cervical cancer screening in >250 clinics in 11 countries in Africa (*21*). PEPFAR cervical cancer screening has become part of a larger public–private partnership known as Pink Ribbon Red Ribbon (PRRR). CDC serves on the steering committee of PRRR, and on the ground, CDC offices in Tanzania, Zambia, Botswana, and Ethiopia collaborate with PRRR in implementation of screening and vaccine services.

For >20 years, CDC has been providing cervical cancer screening to low-income, uninsured, and underserved women in the United States through the National Breast and Cervical Cancer Early Detection Program. Known as one of the few organized screening programs in the United States, this program operates under a set of fundamental tenets that include providing screening and patient navigation services to women for appropriate follow-up and care; quality assurance, surveillance, and monitoring systems by using existing infrastructure help to monitor timeliness and quality of the screening services; and public education and outreach for providers and women to ensure that services are accessed. CDC shares its expertise in operating an organized cervical cancer screening program with other countries by providing technical assistance in the implementation and improvement of cervical cancer screening. Since 2013, CDC has worked with the Thai Ministry of Public Health and the Thai National Cancer Institute to examine strategies to increase cervical cancer screening coverage. CDC and other international partners provided scientific expertise and training for a demonstration project examining the efficacy, feasibility, and cost-effectiveness of primary HPV testing for cervical cancer screening in 1 province in Thailand. The project found that primary HPV testing was feasible and more sensitive than routine Pap-based screening in detecting cervical precancers (*22*). CDC continues to work with the Thai National Cancer Institute in planning for the development of recommendations to expand primary HPV testing for cervical cancer screening in Thailand.

The National Breast and Cervical Cancer Early Detection Program of CDC has also been working with the US-Affiliated Pacific Islands, including freely associated states such as the Federated State of Micronesia, to examine resource-appropriate ways to increase cervical cancer screening coverage. Together with experts from the Office of Population Affairs (Title X), the American Congress of Obstetricians and Gynecologists Committee on Healthcare for Underserved Women, and the American Society for Colposcopy and Cervical Pathology, CDC has organized expert meetings to discuss the possible use of primary HPV testing or visual inspection with acid to increase screening coverage in the Pacific Islands. These methods might enable screening and treatment to take place in 1 or 2 visits with fewer resources than are needed by Pap-based screening. CDC continues to work with health professionals in the US-Affiliated Pacific Islands and with other health organizations in designing a demonstration project that will study the effectiveness of visual inspection with acid or HPV testing in increasing cervical cancer coverage in the region.

### Evaluation of Cervical Cancer Screening Programs

CDC is a partner in the Improving Data for Decision Making in Global Cervical Cancer Program (IDCCP), a project led by the CDC Foundation and aimed at increasing the availability and quality of data for planning and decision-making to improve cervical cancer screening programs in low- and middle-income countries. The IDCCP toolkit was developed with funding from the Bill and Melinda Gates Foundation and offers standardized and globally endorsed guidance that can be adapted at the country level to support the collection of high-quality data for cervical cancer screening programs. WHO and the George W. Bush Institute are key partners in the development of the IDCCP toolkit (*23**,**24*).

The IDCCP tool kit includes 5 modules. The first module is the cervical cancer data systems assessment module that provides guidance on implementing data systems for cervical cancer prevention and treatment. The second is the population-based survey module that provides country stakeholders with standardized questions on cervical cancer screening and treatment that can be incorporated into existing population-based surveys. The third is the cervical cancer patient and program monitoring module that outlines a process for data collection, aggregation, analysis, and reporting for screening and treatment programs. The fourth is the facility-based surveys module that provides tools to gather and evaluate information on location and readiness of facilities to deliver cervical cancer screening and treatment services. The fifth is the comprehensive cervical cancer costing analysis module that enables health program planners to estimate and analyze program and service costs. CDC has been engaged in development and pilot testing of the modules; the modules are under review with partner organizations before final publication (*25*).

CDC also provides technical assistance to countries in analysis of national cervical cancer screening data. CDC collaborated with the Thailand Ministry of Public Health to analyze national cervical cancer screening data from its Behavior Risk Factor Surveillance Survey in 2010 (*26*). CDC also provided technical assistance in analyzing national cervical cancer screening data from China in 2010, conducted an environmental scan in Kenya to assess perceptions of barriers and benefits of cervical cancer screening, and is currently collaborating with the Public Health Foundation of India to assess national cervical cancer screening coverage (*27**,**28*).

## Workforce Development for Global Cancer Prevention

Most cancer cases and deaths occur in low- and middle-income countries, where workforce capacity and resources for cancer prevention and control are limited. Few open-access materials are available to deliver cancer trainings in low-resource settings. To help address this gap, CDC has developed a cancer curriculum for Field Epidemiology Training Programs (FETPs), ministry of health staff, and public health personnel in low- and middle-income countries.

The FETP is a 2-year training in applied epidemiology modeled after the CDC Epidemic Intelligence Service program. The establishment of FETPs started in 1975, and CDC currently supports FETPs in 65 countries (*29*). FETP fellows are primarily trained to respond to public health emergencies from infectious diseases to collect, analyze, and interpret public health data and turn the results into action. In 2010, FETPs started focusing on training field epidemiologists in NCDs to address the growing burden of these diseases in low- and middle-income countries. The CDC cancer curriculum will provide FETPs with applied cancer epidemiology training focused on cancer screening, registration, and comprehensive cancer control. CDC has held workshops to pilot test the curriculum at FETP meetings in Atlanta, India, Nigeria, and Morocco, and the curriculum modules are under review before final publication.

CDC also provides support and mentorship to FETPs in conducting applied research projects. Since 2016, CDC has supported 11 cancer-related FETP research projects, most of which focused on evaluation of programs or analysis of survey data on cervical cancer screening. Some examples of these FETP projects include an assessment of healthcare providers’ knowledge of national guidance for cervical cancer screening in primary healthcare centers in Mexico; an evaluation of the referral mechanism in cervical cancer screening programs in India; and an evaluation of healthcare workers’ knowledge, attitudes, and practices in cervical cancer screening programs in Thailand.

## Improvement of Global Cancer Surveillance

WHO has developed a Global Monitoring Framework on Noncommunicable Diseases to track global progress in preventing and controlling NCDs. All governments have approved this framework, which includes monitoring 3 key indicators related to cervical cancer prevention: national coverage of cervical cancer screening, HPV vaccination, and cancer incidence. Thus, cervical cancer surveillance involves monitoring national screening and HPV vaccination coverage through national data systems and tracking cancer incidence with cancer registries. However, the number of low- and middle-income countries with available estimates of national cancer burden is limited; the percentage of the population covered by cancer registries is estimated to be 2% in Africa, 6% in Asia, and 25% in Latin America and the Caribbean (*30*).

CDC is a key partner in the Global Initiative for Cancer Registry Development that is being led by the WHO International Agency for Research on Cancer (IARC) to improve cancer registration worldwide (*31*). The Global Initiative for Cancer Registry Development aims to increase global capacity to collect high-quality population-based cancer registry data in >150 countries by developing regional hubs that can tailor training and support to countries in that region and also assist in advocacy for cancer registration. CDC provides support for the operation of cancer registry hubs in sub-Saharan Africa and Asia. In addition, CDC is also supporting the newly formed Caribbean cancer registry hub in collaboration with IARC, the US National Cancer Institute, the North American Association of Central Cancer Registries, and the Caribbean Public Health Agency.

CDC has developed a tool to estimate costs of operating population-based cancer registries and has partnered with WHO/IARC to pilot test this tool in several low- and middle-income countries, including Kenya, Uganda, Colombia, India, and Barbados. From these pilot projects, CDC and partners found that the cost for cancer registration for a single cancer case varied from ≈$4 to $113, which translated to only a few cents per person when examined at the population level (*32*). CDC continues to work with several countries to evaluate drivers of costs for cancer registration and to work with additional countries in assessing costs of cancer registration.

## Conclusions

The global burden of cervical cancer remains high, particularly in low- and middle-income countries. Research advances have identified HPV as the cause of nearly all cervical cancers, as well as some cancers of the vagina, vulva, penis, anus, and oropharynx. HPV is an emerging infectious threat; countries can reduce the burden of HPV-associated cancers, including cervical cancer, by implementing HPV vaccination. HPV tests are also a major component to improving cervical cancer screening. CDC provides technical assistance and collaborates with global partners on HPV vaccination and cervical cancer screening. Activities in global cervical cancer prevention build on and further leverage the global footprint of CDC preventing infectious diseases. These activities also relate to the GHSA action packages to improve immunization, surveillance, and public health workforce globally, which contribute to rapid detection, response, and containment of public health emergencies at their sources for enhanced global health security.
